# Type 1 pediatric tympanoplasties using fascia and cartilage grafts^☆^

**DOI:** 10.1016/j.bjorl.2016.09.006

**Published:** 2016-10-22

**Authors:** Zheng-cai Lou

**Affiliations:** Wenzhou Medical University, YiWu Hospital, Department of Otorhinolaryngology, YiWu city, Zhejiang province, China

Dear Editor,

Here, we present a review of the article entitled “Comparison of temporalis fascia muscle and full-thickness cartilage grafts in type 1 pediatric tympanoplasties” by Yegin et al.^1^ The work described in their paper was interesting. Cartilage grafts improved the long-term success rate of tympanoplasties versus temporalis fascia grafts in tympanoplasties due to Eustachian tube dysfunction in children. However, the study design and short follow-up weakened their conclusions.

The authors stated that “A retrospective review of data collected from January 2013 to September 2014 was performed at our hospital” and “The patients were randomly allocated to surgery using temporalis fascia muscle or tragal cartilage grafts by the surgeons” in their Methods.^1^ We believed that a “retrospective review” and “randomly allocated” are contradictory. Randomized control cannot be performed in a retrospective study. Randomized controlled trials, considered the gold standard of study design, are prospective studies. They can provide evidence of cause-and-effect relationships and support changes in clinical practice or workplace interventions. In a randomized controlled trial, subjects are randomly assigned to receive the intervention or control treatment, and outcomes are evaluated after the intervention period. The control group is the group that receives standard care, no intervention, or a placebo. If the patients were not randomized, the results might include some bias. Thus, we believe that the study needs to be a prospective study, with random control, a larger sample size, and longer follow-up to reach valid conclusions.

The authors only excluded patients with ossicular chain defects, cholesteatoma, tympanosclerosis, atelectasia, and a history of previous ear surgery in their criteria. The authors did not state whether patients with granulation tissue in the middle ear were included. Granulation tissue may affect the success of pediatric tympanoplasties, especially for temporalis fascia grafts. Additionally, they reported that the graft success rate was 92.1% for the cartilage group versus 65.0% for the temporalis fascia group during the first postoperative year.^1^ Their success rate in the temporalis fascia group seems very low. There are many reported success rates of >80% in temporalis fascia type 1 tympanoplasties.^2^, ^3^ Although Cabra and Monoux reported a success rate of 64.6% in a fascia group and 82.26% in a cartilage group, their follow-up time was 24 months.^4^ Experimental studies have shown that temporalis fascia grafts can suffer degeneration and shrinkage over time, resulting in reperforation.^5^ Additionally, the Eustachian tube has a significant role in the success of myringoplasty. Two studies in children alone showed better morphological outcomes with the use of cartilage than with fascia grafts. One of the effects of Eustachian tube dysfunction in the pediatric population is negative pressure in the middle ear cavity, which can cause retraction of the tympanic membrane, with resulting failure of myringoplasty.^2^ The authors should evaluate the Eustachian tube in future studies.

The authors stated that the tragal cartilage was harvested together with perichondrium on both sides. An inferior cut was made as low as possible to obtain all of the tragal cartilage in the Methods. Although cartilage graft tympanoplasties may obtain a higher success rate, the excision of all of the tragal cartilage would have esthetic effects in the ears of children. Tragal perichondrium tympanoplasties have certain advantages including higher success rates and no effect on esthetics. Thus, the authors should compare the success rates of type 1 pediatric tympanoplasties using fascia grafts, cartilage grafts, and perichondrium grafts in future work.

## Conflicts of interest

The author declares no conflicts of interest.
